# SOLFA study: a multicenter, open-label, prospective, randomized study to investigate the clotting propensity of asymmetric cellulose triacetate membrane compared to synthetic membranes in on line HDF

**DOI:** 10.1007/s40620-024-02197-y

**Published:** 2025-01-19

**Authors:** Marta Puerta, María Teresa Jaldo, Patricia Muñoz, Patricia Martínez-Miguel, Francisco Maduell, Carolina Lancho, Antonio Luis García-Herrera, Sunny Eloot, Patricia de Sequera

**Affiliations:** 1 Department of Nephrology, University Hospital Infanta Leonor, Madrid, Spain; 2 Department of Nephrology, Hospital Universitario Príncipe de Asturias, Madrid, Spain; 3 Department of Nephrology, Hospital Clinic, Barcelona, Spain; 4 Department of Nephrology, Hospital Puerto Real, Cádiz, Spain; 5 Department of Nephrology, Ghent University Hospital, Ghent, Belgium; 6 Department of Medicine, Universidad Complutense de Madrid, Madrid, Spain; 7 RICORS2040 Madrid, Spain

**Keywords:** Anticoagulation, Heparin, Biocompatibility, Hemodialysis

## Abstract

**Background:**

Performing hemodialysis without heparin is still challenging. The objective of the present work was to evaluate the impact on thrombogenicity of the hemodialysis circuit using synthetic membranes compared to the asymmetric cellulose triacetate** (**ATA) membrane.

**Methods:**

Prospective, multicenter, randomized, crossover, open-label study. In each of the two phases of the study, six consecutive hemodialysis sessions were performed over two weeks, in which the patients were dialyzed with the dialyzer randomly assigned (synthetic vs asymmetric cellulose triacetate membrane). During the six sessions of both phases, the heparin dose was progressively reduced from the full usual heparin dose in the first session to zero heparin in the sixth session. After each session, visual inspection of the venous chamber and dialyzer was performed, and a coagulation score was assigned. A micro- computed tomography (CT) scanning of some dialyzers was also executed at Ghent University.

**Results:**

Comparison of the last completed sessions shows that there were significant differences depending on the dialyzer used: 60% of dialysis sessions with asymmetric cellulose triacetate could be completed without heparin versus 24% with synthetic membranes (*p* = 0.01). We also found differences in the number of sessions completed: 46% with the asymmetric cellulose triacetate membrane and 7% with the synthetic membrane (*p* = 0.001). The results obtained with the micro-CT analysis were also better with the asymmetric cellulose triacetate.

**Conclusions:**

Our findings strongly suggest that asymmetric cellulose triacetate membranes may be useful in situations in which dialysis should be performed without heparin or with low-dose heparins.

**Trail registry:**

NCT06505616.

**Graphical abstract:**

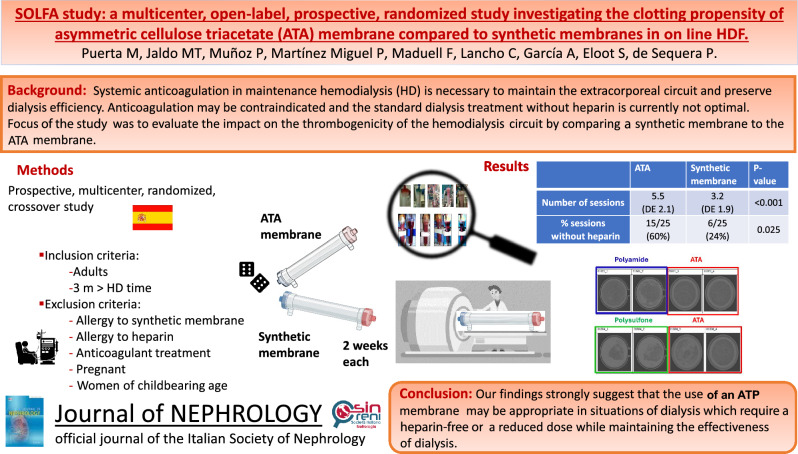

**Supplementary Information:**

The online version contains supplementary material available at 10.1007/s40620-024-02197-y.

## Introduction

Systemic anticoagulation is necessary during hemodialysis (HD) to preserve the extracorporeal circuit and maintain dialytic efficacy [1]. Both low molecular weight heparin (LMWH) and unfractionated heparin (UFH) are used in dialysis units in Spain. Although European guidelines recommend using LMWH [2], several studies have shown the same efficacy and safety for both types of heparin [3]. Inadequate heparinization likely results in coagulation of a considerable number of fibers, with the consequent worsening of dialytic parameters, affecting both diffusive and convective transport [4].

The objective of heparinization in dialysis is to use the minimum dose of heparin required to prevent coagulation of the extracorporeal circuit but also to avoid complications such as cerebral or retinal hemorrhage, vascular access bleeding, and gastrointestinal hemorrhage, among others [5]. In addition, the best way of performing hemodialysis with low-dose or no heparin has not yet been established.

Anticoagulation may be contraindicated in certain situations such as in patients with active bleeding or at high risk of bleeding, with allergy or heparin-induced thrombopenia. In these circumstances, different strategies have been developed to maintain the patency of the circuit without the administration of heparin, such as regional anticoagulation with citrate, administration of direct thrombin inhibitors, and intermittent saline flushes [6, 7], dialysis fluid with citrate [8], and modified dialysis membranes such as heparin-coated membranes [9, 10]. Currently, dialysis treatment without heparin is not optimal and more effective methods are needed to prevent clotting in the extracorporeal circuit.

Several factors can increase the risk of circuit and dialyzer clotting, including low blood flow, high hematocrit, high ultrafiltration rate, or technical errors. [11]. The influence of the type of treatment on the coagulation system may also play a role. Although post-dilution online hemodiafiltration (HDF) improves intradialytic hemodynamic tolerance and increases survival [12, 13], it is associated with a greater procoagulant effect as compared to high-flux hemodialysis. Among other factors, a procoagulant effect is due to hemoconcentration in the dialyzer, and heparin clearance is increased in convective techniques [14]. Other factors that may influence the risk of coagulation are the characteristics of the membrane, including electrical charge, chemical composition, and the ability to activate the coagulation cascade [15].

Some synthetic membranes used in HDF may occasionally induce hypersensitivity reactions during dialysis sessions. The cause of these reactions remains uncertain, but they disappear after replacing the membrane, usually with one made of cellulose triacetate [16]. In recent years, a new type of cellulose triacetate membrane has been developed, i.e., the asymmetric cellulose triacetate (ATA™) membrane (Solacea™, Nipro, Osaka, Japan). This membrane has not only demonstrated similar efficacy in achieving the objectives of HDF compared to synthetic membranes [17], but has also been shown to have a lower propensity for circuit coagulation, even with low doses of heparin [18, 19].

The main objective of the present study was to evaluate the impact of the asymmetric cellulose triacetate membrane on thrombogenicity of the hemodialysis circuit as compared to other commonly used high-permeability synthetic membranes. The primary endpoint was the number of sessions performed with a reduced dose of heparin and the number of sessions completed without heparin.

Secondary objectives were to evaluate the effect of reducing or discontinuing heparin on vascular access hemostasis time and dialysis efficiency by measuring: Kt, percentage reduction of molecules such as myoglobin or β2-microglobulin, and in the amount of convective volume in patients on HDF.

## Methods

### Study design

This was a prospective, multicenter, randomized, crossover, open-label study that included 32 patients from 4 different hospitals in Spain: University Hospital Infanta Leonor and Príncipe de Asturias (Madrid), Puerto Real (Cádiz) and Clinic (Barcelona).

### Study population

All patients were over 18 years of age, in a chronic hemodialysis program for at least three months, and gave informed consent. The study was approved by the local Ethics Committee and was conducted in accordance with the principles of the Declaration of Helsinki. Exclusion criteria were allergy to synthetic membranes and/or heparin, patients on anticoagulant treatment, pregnant or breastfeeding women, and patients with cognitive impairment.

### Treatment procedures

**Fig. 1:**
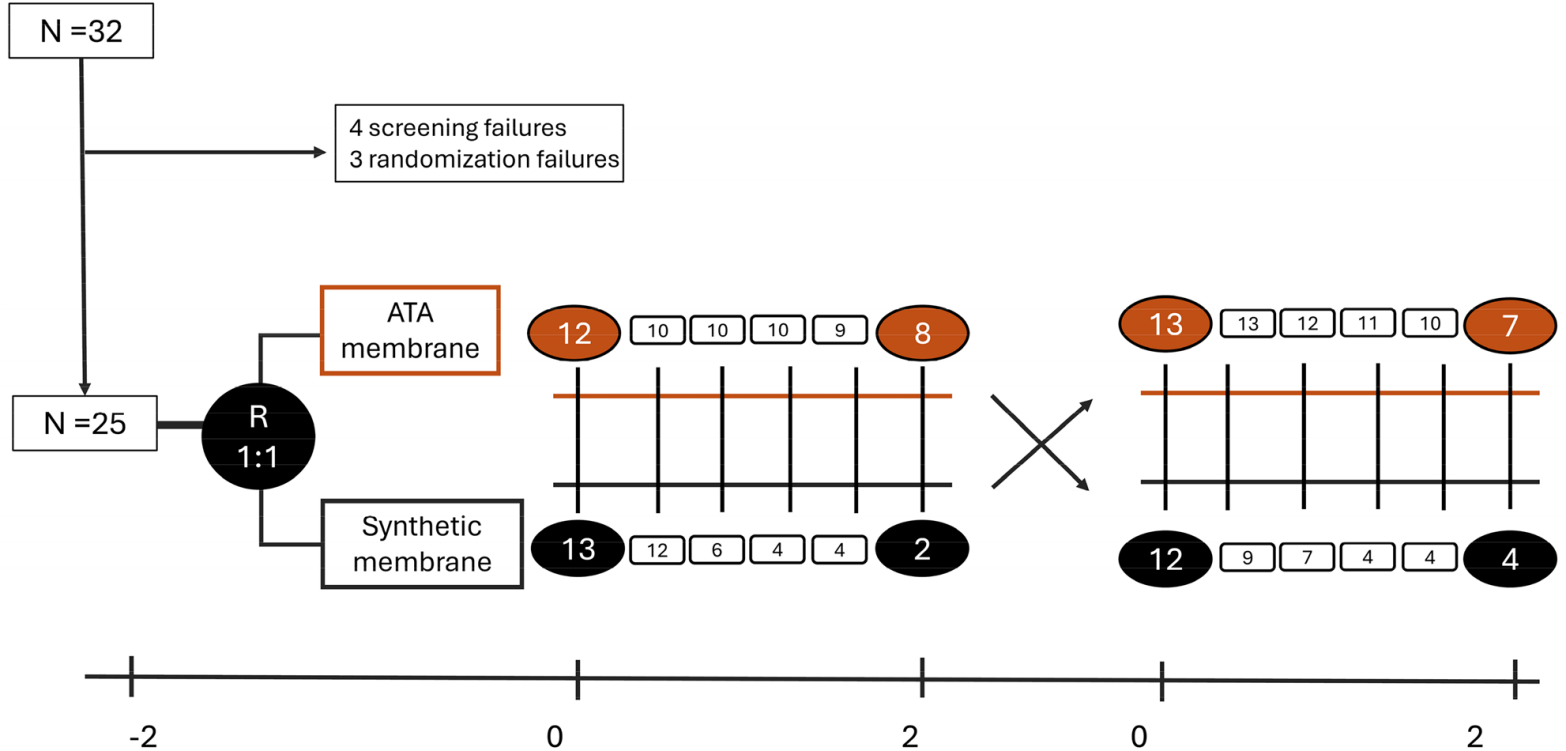
Study design and flow chart.

The duration of the study was four weeks, and it was carried out in two phases. After selection, patients were randomized to dialysis with the asymmetric cellulose triacetate membrane versus the usual dialyzer (Fig. 1), which in all cases had to be a high-flux synthetic membrane. In the first phase, patients were dialyzed for two weeks according to their usual regimen with the dialyzer assigned after randomization. Table S1 shows the in vitro characteristics of the dialyzers used in the study.

All on line HDF sessions were in post-dilution hemodiafiltration mode. Dialysis sessions were not standardized in the four centers, but they had similar dialysis conditions with the same dialyzers, similar dialysis treatment time and blood flows. Aside from the dialyzer and heparin dose, no other treatment settings were changed during the study, and the patients were their own internal controls in this crossover study. The characteristics of the dialysis sessions are described in the supplementary material (Tables S2 and S3).

In the second phase, also lasting two weeks, patients were crossed over to the other group, that is, patients assigned in the first phase to the asymmetric cellulose triacetate membrane were dialyzed with their usual dialyzer in the second phase, while patients first assigned to their usual synthetic dialyzer were dialyzed with the asymmetric cellulose triacetate membrane in the second phase (Fig. 1). During the six sessions of both phases, the heparin dose was progressively reduced until being heparin-free in the last session (Supplementary Material).

After each session, two people from the nursing team carried out a visual inspection of the venous chamber and the dialyzer and assigned a coagulation score (Figure S1) as previously published [20]. If there was no unanimity between the two, a third person from the nursing team proceeded to evaluate the score. If the dialyzer score was 3–4, or the venous chamber score was 2–3, the heparin dose could not be tapered any further, and that session was the last.

If the session ended prematurely due to the risk of coagulation of the hemodialysis circuit, with the need to use intermittent saline flushes, or coagulation of the dialyzer, or if venous chamber occurred, it was considered the end of the of the study. Baseline demographic data and dialysis parameters were also collected (Table S2-S3). Blood samples for analyses were taken from each patient at the same week's dialysis session (Tables S4-S5). From the principal investigator's center, dialyzers were collected from 8 patients at the beginning and end of each study arm and analyzed with a noninvasive micro-computed tomography (CT) scanning technique at Ghent University. [21]. They tested the reproducibility and the effect of the chosen cross-section in which they measured the number of permeable fibers. They found a correlation with the mass dialyzer after dialysis; there were correlations between the number of permeable fibers and the dry mass of dialyzers after dialysis (*R*^2^ = 0.62). A total of 32 dialyzers of 3 different types were analyzed by micro-CT: FXCorDiax800 (6 + 6), Solacea-21H (8 + 8) and Polyflux 210H (2 + 2). In those patients who did not reach the end of the study due to coagulation of the circuit, the dialyzer from the last session was collected. In summary, at the end of the dialysis session, a standard procedure of washing the dialyzer with 300 ml of washing solution was performed. The dialyzer was then dried for 24 h using continuous mild positive pressure ventilation simultaneously in the blood and dialysate compartment. The coagulation of the dialyzer fibers was visualized in the dialyzer output shell using a 3D CT scanning technique with micrometer resolution, described previously [18]. For this study, three different thresholds were used to define the surface area of an open fiber: i.e. 50%, 70% and 90% of the cross-section of a non-used fiber. Comparison of the number of non-coagulated fibers in the tested dialyzer with the total number of fibers measured in three samples of non-used dialyzers, provided an objective estimate of the percentage of coagulated fibers.

The impact of the study is not limited by the small number of patients because by using a cross-over design each patient serves as his or her own control.

## Statistical analysis

Statistical analysis was performed using Stata v14 (StataCorp. 2015. Stata Statistical Software: Release 14. College Station, TX: StataCorp LP) A p-value less than 0.05 was considered significant.

Continuous variables are shown as median and interquartile range (IQR), and categorical variables as percentages. The Kolmogorov–Smirnov test was used to determine whether the data were normally distributed and the Levene test to evaluate heterogeneity. Data were analyzed by Chi-Square, paired t-Test, One-way ANOVA and Tukey tests.

## Results

### Baseline data

Of the 32 patients initially recruited, 7 were excluded, of whom 4 did not meet all the inclusion criteria and 3 because of randomization failure (Fig. 1).

The usual technique was on line HDF in 21 (84%) and conventional hemodialysis in 4 (16%). The usual dialyzers are listed in table S1. In the subgroup for micro-CT scanning technique, only FX_CorDiax_ 800 and Polyflux 210H were used as synthetic dialyzers, as well as the Solacea-21H.

The mean age of the patients was 70.1 years, and 60% were men. The etiology of kidney disease was: 16% glomerular, 20% vascular, 20% diabetes, 12% interstitial, 4% polycystic disease and 28% unknown. Fifty-two percent of the patients were diabetic and their mean + SD body mass index (BMI) was 24.6 + 3.4) kg/m^2^. On average, the patients had started kidney replacement therapy (KRT) 2 years earlier (1.1–7.8 years), 17 (68%) were dialyzed using their native arteriovenous fistula (AVF), 4 (16%) had a synthetic arteriovenous graft (AVG), and 4 (16%) had a central venous catheter (CVC). The usual technique was on line HDF in 21 patients (84%) and conventional hemodialysis in 4 (16%). The usual dialyzers and their characteristics are listed in Table S1. In the subgroup of dialyzers that underwent the micro-CT technique, only FXCorDiax 800 and Polyflux 210H were used as synthetic dialyzers, while the Solacea-21H was used for the asymmetric cellulose triacetate membrane.

### Dialysis efficiency and completion of the dialysis sessions

 There were no statistical differences between the dialysis data obtained with asymmetric cellulose triacetate and those obtained with the synthetic membranes during the first dialysis session with the full dose (100%) of heparin. The data are presented in supplementary Table 2. The data obtained during the last dialysis session are shown in table S3.

The use of the asymmetric cellulose triacetate membrane allowed a greater number of sessions to be carried out without heparin (15/25, 60%) than with the usual synthetic membrane (6/25, 24%) (*p* = 0.025). (Table 1). We also observed a significant difference in the number of sessions performed with a reduction of the heparin dose: 46% with the asymmetric cellulose triacetate membrane vs 7% with the synthetic membrane, *p* = 0.01.

**Table 1:** Number of dialysis performed in each session according to dialyzer

	ATA membrane	Synthetic membrane
1st session 100% heparin (N, %)	25 (100%)	25 (100%)
2nd session 75% heparin (N, %)	23 (92%)	21 (84%)
3rd session 50% heparin (N, %)	22 (88%)	13 (52%)
4th session 50% heparin (N, %)	21 (84%)	8 (32%)
5th session 25% heparin (N, %)	19 (76%)	9 (32%)
6th session 0% heparin (N, %)	15 (60%)	6 (24%)

These results are in line with the coagulation score obtained in the dialyzers. A coagulation score of 3–4 of the dialyzer was found in only 18% of the asymmetric cellulose triacetate membrane sessions, while it was reported in 60.7% of sessions with synthetic membranes (*p* = 0.04).

The average number of sessions completed following the heparin reduction scheme established in the protocol also differed with the use of the asymmetric cellulose triacetate membrane vs synthetic membranes. The average number of sessions completed (out of 6 sessions), with the asymmetric cellulose triacetate membrane was 5.3 + 2.1, while in the case of synthetic membranes it was 3.3 + 2 (*p* < 0.001), which means that more sessions could be completed with the asymmetric cellulose triacetate membrane despite reducing the dose of heparin.

Dialytic efficacy, estimated from the Kt of the sessions and the percentage reduction of β2-microglobulin, was similar between sessions, and was neither affected by the heparin reduction nor by the type of dialyzer.

No significant changes were observed in the analytical measurements performed pre- and post-dialysis, except for the post-dialysis myoglobin values which were lower with the asymmetric cellulose triacetate membrane: 45.2 + 12.7 ng/ml compared to the synthetic dialyzers: 90 + 33.9 ng/ml *p* = 0.003.

### Clotting of the dialyser 

**Fig. 2:**
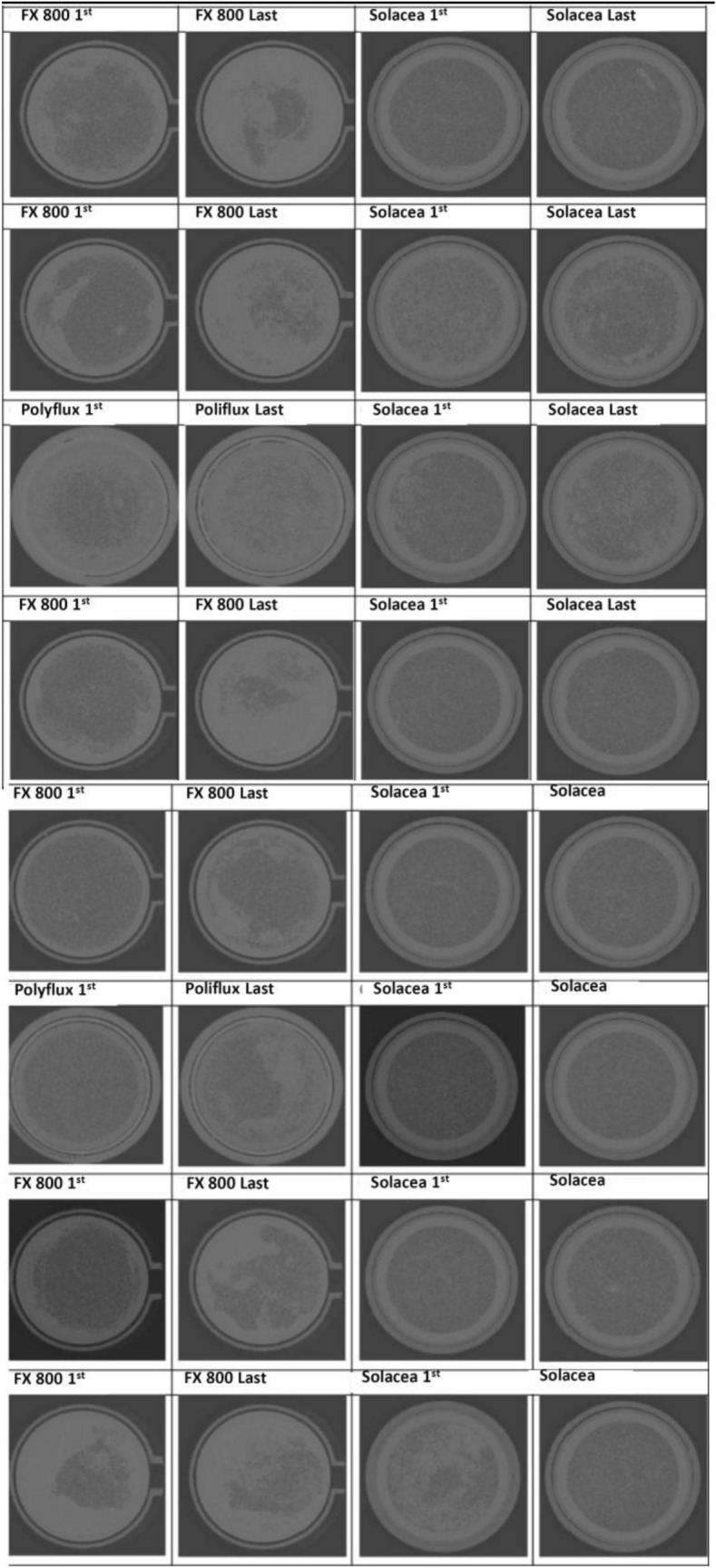
Reconstructed images of the cross-section micro-CT for dialyzers used in the first and last sessions of 8 patients

The frequency of dialyzers that have more than 50% of open fibers is shown in Table 2 and Fig. 2. The data indicate the limits at which the fiber is considered open (50, 70 and 90%). There were no differences between the results at the limits of 50, 70 and 90% for each dialyzer. But there were differences between the different types of dialyzers for each of the limits.

**Table 2:** Percentage of open fibers in the dialyzers as assessed by the micro-CT for the limits of 50, 70 and 90% open fiber area

		Polysulfone (FX_CorDiax_ 800)		Polyamide (Polyflux 210H)		ATA membrane (Solacea-21H)	
	Session	1st	Last	1st	Last	1st	Last
	N	6	6	2	2	8	8
50% open area	Median	80%	33.3^#%^	74.8%	45.15^#%^	98.6%	96.1^#%^
	IQR range	72.8–81.9	23.7–55.4	50.1–99.5	31–59.3	74.95–99.45	82.4–98.6
70% open area	Median	79.45%	32.65^#%^	74.25%	43.2^#%^	97.7%	95.35^#%^
	IQR range	71.7–81.7	23.1–52	49.1–99.4	27.6–58.8	74.6–99.05	81.8–98.35
90% open area	Median	49.7%	14.95*%	64.65%	31.7*%	72.45%	76.15*%
	IQR range	41.1–53.2	9.1–19.6	42.5–86.8	14.3–49.1	58.55–77.85	60.55–81.15

*p* value *0.003 y # 0.005

By comparing the sessions independently, it can be seen that in the first sessions the results are similar between dialyzers (*p* = 0.4 for 50%; *p* = 0.1 for 70% and *p* = 0.6 for 90%). However, in the lowest heparin dose session, differences are observed in all thresholds 50% (*p* = 0.02), 70% (*p* = 0.02), and 90% (*p* = 0.002), with results obtained by the asymmetric cellulose triacetate membrane being more favorable in all cases.

The analysis between the first and last session for each of the dialyzers reflected their different performance. While the asymmetric cellulose triacetate membrane maintained a similar range of open fibers between the first and last session (*p* = 0.7 at 50%; *p* = 0.8 at 70% and *p* = 0.3 at 90%), in FX_CorDiax_800 membranes the number of open fibers was lower in the last session (*p* = 0.02 at 50%; *p* = 0.02 at 70% and *p* = 0.02 at 90%).

## Discussion

The present randomized crossover study investigated the impact of the asymmetric cellulose triacetate membrane versus synthetic dialyzers on the thrombogenicity of the hemodialysis circuit. The number of sessions completed without or with reduced heparin dose were analyzed. Coagulation was detected by two methods: firstly using a visual scale with a coagulation score that included both the dialyzer and the circuit chambers, and secondly by measuring the number of obstructed fibers using micro-computerized tomography.

One of the strengths of the study is that it focuses on online hemodiafiltration, while previous studies mostly focused on hemodialysis.

In order to avoid or reduce anticoagulation in hemodialysis, some guidelines [22–24] and experts [25] recommend using saline flushes and predilution hemodiafiltration. Dilution of the blood entering the dialyzer, as in predilution hemodiafiltration, is an effective method to reduce coagulation because it reduces concentrations of platelets and clotting factors as well as the viscosity by reducing the hematocrit, thus lowering the risk of clotting. However, in the present study we explored the effect of dialyzers in post-dilution hemodiafiltration because this is the most widely used method of hemodiafiltration in Europe and the one that provides the best results [12, 13].

There are two main findings of this study; the first was that the number of sessions completed with a reduced heparin dose or without heparin was higher with the asymmetric cellulose triacetate membrane than with commonly used synthetic membranes. Of the sessions completed without heparin, 60% involved the asymmetric cellulose triacetate membrane and 24% involved synthetic membranes, *p* = 0.02. In the last session, those dialyzed with an asymmetric cellulose triacetate membrane were able to use 43.8% of the usual heparin dose, while the group with the synthetic membrane had to use 75% of the usual heparin dose. Even with a greater reduction of heparin, the asymmetric cellulose triacetate membrane showed fewer coagulated fibers than the usual synthetic membranes. The heparin reduction achieved with the asymmetric cellulose triacetate membrane did not translate into adverse effects on dialysis efficiency measured by Kt or in the convective volume achieved in patients treated with HDF.

A second relevant finding of this study was the lower number of clotted fibers with the asymmetric cellulose triacetate membrane as compared to the synthetic membranes, determined both by visual score and by the micro-CT analysis. A 3–4 dialyzer coagulation score was obtained in only 18% of sessions with asymmetric cellulose triacetate membrane, while it was as high as 60.7% with synthetic membranes (*p* = 0.04). The proportion of open fibers post-dialysis was greater with the asymmetric cellulose triacetate membrane (Solacea-21H) than with synthetic membranes (FX_CorDiax_800 and Polyflux 210H). These values were obtained by using micrometric techniques of 3D computed tomography (3D micro-CT) in a subgroup of 8 patients (32 dialyzers in total). This method is much more sensitive than visual inspection of the dialyzer. Therefore, the 3D micro-CT method confirmed the observation that the asymmetric cellulose triacetate membrane presents a greater number of open fibers than synthetic dialyzers. These results are in line with other studies that evaluated the thrombogenicity of asymmetric cellulose triacetate membranes. The SAFE study [18] compared patients with an asymmetric cellulose triacetate membrane and high volumes of predilution hemodiafiltration with patients on dialysis with citrate, resulting in a high rate of dialysis termination without filter coagulation, in both groups. Other investigators compared asymmetric cellulose triacetate membranes against other dialyzers. Vanommeslaeghe et al. compared the thrombogenicity of the asymmetric cellulose triacetate membrane with that of FX_CorDiax_800 polysulfone dialyzer, evaluating the resistance to fiber blockage using micro-CT scanning [26], demonstrating superiority of asymmetric cellulose triacetate membrane in the relative number of post-dialysis fiber permeability, independently of the anticoagulation dose.

The asymmetric cellulose triacetate membrane demonstrates superior antithrombotic properties as compared with synthetic membranes that have long been used. It is thought that the reduced protein adsorption to the cellulose triacetate membrane is partly responsible for its antithrombotic properties [27]. The electrostatic properties arising from the chemical structure of cellulose triacetate and the smoother surface of the membrane have been identified as the factors responsible for less protein adsorption [28]. Cellulose triacetate and asymmetric cellulose triacetate are made of the same material, the difference is that asymmetric cellulose triacetate has an asymmetric structure and less irregularities or roughness in the inner surface of the membrane [29].

It is important to highlight the need for studies that provide evidence on the topic of heparin-free treatments. The reason is that in the majority of hemodialysis facilities, there are no circuit anticoagulation protocols. Practice is based on the unrestricted use of anticoagulants, which can lead to complications due to both insufficient or excessive use, since the needs are highly variable, depending both on the patient's factors and on the dialysis technique [5]. Furthermore, it is a priority to have alternatives for those patients with active bleeding or increased risk of bleeding, for whom anticoagulation needs to be avoided and other methods are used, like saline flushes, predilution HDF, dialyzers with a heparin-coated membrane, or citrate-based dialysate.

The main limitation of our study is the small number of included patients, but the randomized and crossover design meant that each patient was his own internal control, thus reinforcing the strength of the results. A second constraint was the use of heparin dose reduction percentages because patients had UFH and LMWH interchangeably. A third limitation of the study is that the scanning technique was carried out only in a few dialyzers, i.e., asymmetric cellulose triacetate, polysulfone and polyamide; these synthetic membranes are commonly used in HDF in the hospitals included in our study. Another drawback of the design could be that visual inspection is not a fully reliable method to assess the clotting of the fibers, although this was partially solved by performing micro CT in a representative sample of dialyzers.

Furthermore, it would have been interesting to include a heparin-coated (Evodial) dialyzer in the study, which is recommended by some clinical practice guidelines and commonly used in some dialysis units, when reducing or withdrawing heparin is necessary.

Our results, although of potential interest, cannot be generalized due to the use of the high blood flows and post-dilution hemodiafiltration mode. Nevertheless, both flow and technique were the same for asymmetric cellulose triacetate and synthetic membranes.

Probably the best approach for performing hemodialysis without heparin includes the combination of different strategies such as citrate-buffered dialysate, predilution hemodiafiltration and filters having a low propensity for coagulation.

## Conclusions

Our current findings suggest that the asymmetric cellulose triacetate membrane may be useful in situations in which it is important either to perform the dialysis session without heparin or to reduce heparin dose while maintaining the effectiveness of dialysis.

## Supplementary Information

Below is the link to the electronic supplementary material.

Supplementary file1 (DOCX 148 KB)

## Data Availability

The authors confirm that the data supporting the findings of this study are available within the article and its Supplementary material. Raw data that support the findings of this study are available from the corresponding author, upon reasonable request.
