# Supplementary material for: SOLFA study: a multicenter, open-label, prospective, randomized study to investigate the clotting propensity of asymmetric cellulose triacetate membrane compared to synthetic membranes in on line HDF
Source: J Nephrol. 2025 Jan 19;38(2):697–705. doi: 10.1007/s40620-024-02197-y (PMC11961484; doi:10.1007/s40620-024-02197-y)
Supplement: Supplementary file 1 — Supplementary file1 (DOCX 148 KB) [file 40620_2024_2197_MOESM1_ESM.docx]

**SOLFA study: A multicenter, open-label, prospective, randomized study investigating the clotting propensity of asymmetric cellulose triacetate (ATA**) **membrane compared to synthetic membranes in online HDF.**

**Supplementary material**

**Supplementary methods**

Rationale for the applied heparin dosing reduction regimens

In a previously conducted multicenter study on the usefulness of citrate dialysis fluid that included its effect on anticoagulation [^[[1]](#endnote-2)^], no differences were found in the coagulation scores between acetate and citrate because the heparin dose used was too high. Based on this experience, it was decided that the study would benefit from a design where the heparin doses was progressively reduced until the heparin was completely discontinued. As the goal of this study was to see the effect of dialyzer membranes on coagulation.

During the six sessions of both phases, the dose of heparin was progressively reduced as follows:

- First session: 100% usual heparin dose.
- Second session: 75% of the usual heparin dose.
- Third session: 50% of the usual heparin dose
- Fourth session: 50% of the usual heparin dose. In this session, the dose of heparin was not modified to avoid the effects that the higher ultrafiltration rate could have after a long period of the weekend due to the greater interdialytic weight gain that usually occurs
- Fifth session: 25% of the usual heparin dose
- Sixth session: without heparin

After each session, two people from the nursing team carried out a visual inspection of the venous chamber and the dialyzer and assigned a coagulation score previously published (figure S1). If there was no unanimity between the two, a third person from the nursing team proceeded to evaluate the score. The session was considered to have ended without incident when the visual score of the dialyzer was less than 3 and that of the venous chamber less than 2.

Baseline demographic data were collected: age, sex, aetiology of chronic kidney disease, time on dialysis, dialysis fluid (acetate-citrate) and type of vascular access. In addition, analytical determinations were performed before and after the first session of each arm and before and after the sixth session of each arm, which included: blood count, activated partial thromboplastin time (APTT), prothrombin time (PT), International Normalized Ratio (INR), anti-Xa, creatinine, urea, glucose, sodium, potassium, albumin, prealbumin, total proteins, β2-microglobulin, myoglobin and C‑reactive protein (CRP). If the patient did not complete all the sessions due to circuit coagulation, only data from the first session was collected.

Dialysis parameters were also collected including: effective time, blood flow (Qb), dialysis dose measured by ionic dialysance (Kt), reinfusion volume in patients undergoing OL-HDF, dry weight, interdialytic weight gain, ultrafiltration volume, intra-dialytic hypotension and haemostasis time in patients with arteriovenous fistula (AVF).

**Statistical Analysis**

Data are shown as mean (standard deviation) or median (interquartile range) if they are quantitative variables or as frequency (percentage) depending on the variables analyzed. The data are compared with the Chi² test or the Kruskal Wallis test.

Comparisons between dialyzers are made with the Student’s t-test for paired samples if it follows a normal distribution (Kolmogorov test) or with the Man Whitney or Kruskal Wallis test if it does not. For qualitative variables, statistical significance is performed with Chi² McNemar.

To evaluate that there was no period effect (the order in which the membrane is evaluated was not relevant for the analysis), nor a carry-over effect (the effect produced by the first membrane during the first phase of the study did not influence the results of the second phase), the average number of viable sessions in each of the two periods were compared and the results indicate that there is no difference between the two periods (Student's t-test, *p*=0.9): G1-G2 1.9 and G2- G1 1.7, *p*= 0.8.

To evaluate the carry-over effect, we compared whether the number of viable sessions is similar in both periods. The Student’s t-test analysis showed that there were no statistically significant differences either (G1-G2: 8.7 vs G2-G1: 7.8, p= 0.4), so we concluded that there was no carry-over effect. Once these points were verified, the study was evaluated jointly, expanding the number of sessions to be analysed.

The entire analysis was done by intention to treat, to maintain what was established in the protocol and not bias the results by introducing subjective elements.

**Supplementary results**

The average haemostasis time per session was 10.3 minutes in the first session, 8.5 minutes in the 4^th^ session (50% heparin) and 10.4 in the last session of the phase, without statistically significant differences, nor differences between dialyzers. Other variables analyzed such as activated partial prothrombin time, thrombin time, INR also did not show differences between sessions or dialyzers.

**Supplementary discussion**

Unfortunately, there was no combination of *in vitro* tests used in the evaluation of membrane thrombogenicity. However, the coagulation before and after the session was analyzed in some patient's blood, platelets, international normalized ratio (INR), partial thromboplastin time (PTT), prothrombin time (PT) and anti-factor Xa activity (Table S6) and no differences were observed depending on the membranes.

**Table S1: *In vitro* features of the dialyzers under investigation.**

|  | Solacea- 21H  (Nipro) | FX_CorDiax_ 800  (Fresenius) | Polyflux 210H  (Baxter) | Revaclear 400  (Baxter) | Elisio -19H  (Nipro) |
| --- | --- | --- | --- | --- | --- |
| Membrane | ATA | PS/PVP | PAES/PVP/Polyamide | PAES/PVP | Polynephron |
| Priming volume (ml) | 118 | 115 | 125 | 93 | 115 |
| KUF* (ml/h/mmHg) | 76 | 62 | 85 | 54 | 76 |
| Wall thickness (µm) | 25 | 35 | 50 | 35 | 40 |
| Inner diameter (µm) | 200 | 210 | 215 | 190 | 200 |
| Surface area (m^2^) | 2.1 | 2.0 | 2.1 | 1.8 | 1.9 |
| Sterilisation method | Dry gamma | Steam | Steam | Steam | Dry gamma |
| SC* β2-microglobulin | 0.85 | 0.9 | 0.82 | 0.95 | 1.02 |
| SC* myoglobin | 0.80 | 0.5 | 0.37 | 0.68 | 0.61 |
| SC* albumin | 0.013 | <0.001 | 0.0022 | 0.0027 | 0.0017 |
| *Presented here are results of *in vitro* tests performed by the different dialyzer manufacturers. Settings of these tests may differ depending on the membrane. KUF: Ultrafiltration coefficient; SC: sieving coefficient; ATA: Asymmetric cellulose triacetate; PS: Polysulfone; PAES: polyarylethersulfone; PVP: polyvinylpyrrolidone. | | | | | |

**Table S2: Data from the first dialysis sessions (100% heparin):**

|  | **ATA membrane** | | | **Usual synthetic membranes** | | |
| --- | --- | --- | --- | --- | --- | --- |
|  | **N** | **Mean** | **SD** | **N** | **Mean** | **SD** |
| Pre dialysis weight (kg) | 25 | 66.1 | 11 | 25 | 66.1 | 11 |
| Post dialysis weight (kg) | 25 | 68.7 | 11.1 | 25 | 68. | 11.4 |
| Dialysis fluid Acetate (%) | 25 | 20 (80%) |  | 25 | 20 (80%) | 11.1 |
| Scheduled time (min) | 25 | 250.2 | 29.6 | 25 | 249.4 | 29.8 |
| Effective time (min) | 24 | 249.3 | 26.4 | 25 | 249.6 | 35.4 |
| Blood flow (Qb; ml/min) | 24 | 395.8 | 55.9 | 25 | 402 | 35.5 |
| Dialysis fluid flow (ml/min) | 25 | 468 | 47.6 | 25 | 468 | 47.6 |
| Reinfusion Volume (l) | 18 | 23.5 | 13.4 | 21 | 22.4 | 10.5 |
| Ultrafiltration volume (l) | 24 | 2.6 | 0.7 | 25 | 2.6 | 1.0 |
| Convective volume (l) | 23 | 27 | 12.4 | 24 | 25.9 | 10.5 |
| Infusion flow (ml/min) | 23 | 98,3 | 48.3 | 22 | 90.5 | 40.4 |
| Kt (l) | 25 | 60 | 12.8 | 25 | 60 | 11 |
| Transmembrane pressure (mmHg) | 25 | 177.2 | 118.8 | 25 | 158.6 | 75.8 |
| Haemostasis time (min)* | 21 | 10.2 | 3.3 | 21 | 10.3 | 2.9 |
| Usual heparin dose (%) | 25 | 100.0 |  | 25 | 100.0 |  |
| Hypotension (%) | 25 | 100.0 |  | 25 | 100.0 |  |
| Completed session (%) | 25 | 100.0 |  | 25 | 100.0 |  |
| Dialyzer Coagulation score | | | | | | |
| 0  1  2  3  4 | 25 | N=8  N=15  N=2  N=0  N=0 | 32%  60%  8%  0  0 | 25 | N=14  N=11  N=6  N=3  N=1 | 16%  44%  24%  12%  4% |
| Coagulation chamber score | | | | | | |
| 0  1  2 | 25 | N=11  N=12  N=2 | 44%  48%  8% | 25 | N=11  N=13  N=1 | 44%  52%  4% |

*Only in arteriovenous fistulas

**Table S3: Dialysis data from the last viable session**

|  | **ATA membrane** | | | **Synthetic membranes** | | |  |
| --- | --- | --- | --- | --- | --- | --- | --- |
|  | **N** | **Mean** | **SD** | **N** | **Mean** | **SD** | **p value** |
| Pre dialysis weight (kg) | 25 | 67.8 | 11 | 25 | 67.9 | 11.3 | 0.98 |
| Post dialysis weight (kg) | 25 | 65.7 | 10.9 | 25 | 65.9 | 11 | 0.96 |
| Dialysis fluid Acetate (%) | 25 | 20 (80%) |  | 25 | 20(80%) |  | 1 |
| Scheduled time (min) | 25 | 250.2 | 29.6 | 25 | 250.4 | 29.8 | 0.98 |
| Effective time (min) | 25 | 243.7 | 31.9 | 25 | 246.3 | 28.1 | 0.8 |
| Blood flow (QB; ml/min) | 25 | 400 | 44.3 | 25 | 392.8 | 44.7 | 0.6 |
| Dialysis fluid flow (ml/min) | 25 | 463,8 | 49.3 | 25 | 468 | 47.6 | 0.8 |
| Reinfusion Volume (l) | 19 | 23.3 | 11.9 | 21 | 23.2 | 11 | 0.98 |
| Ultrafiltration volume (l) | 25 | 2.2 | 0.7 | 25 | 2.2 | 0.8 | 0.8 |
| Convective volume (l) | 24 | 26.1 | 10.8 | 24 | 25.6 | 10.4 | 0.9 |
| Infusion flow (ml/min) | 21 | 100.4 | 44.7 | 20 | 91.2 | 40.8 | 0.5 |
| Kt (l) | 25 | 57.5 | 10.9 | 25 | 58.7 | 10.7 | 0.7 |
| Transmembrane pressure (mmHg) | 25 | 167.8 | 64.7 | 25 | 155.7 | 63 | 0.5 |
| Haemostasia time (min)* | 21 | 10.7 | 2.7 | 21 | 10.6 | 3.4 | 0.95 |
| Usual heparin dose (%)  100  75  50  25  0 | N=2  N=1  N=3  N=4  N=15 | 8%  4%  12%  16%  60% |  | N=4  N=8  N=5  N=2  N=6 | 16%  32%  20%  8%  24% |  | 0.025 |
| Hypotension (%) | 25 | 100.0 |  | 25 | 96 |  | 0.3 |
| Dialyzer Coagulation score | | | | | | | |
| 0  1  2  3  4 | N=0  N=13  N=7  N=5  N=0 | 0%  52%  28%  20%  0% |  | N=0  N=3  N=5  N=10  N=7 | 0%  12%  20%  40%  28% |  | 0.002 |
| Chamber coagulation score | | | | | | | |
| 0  1  2  3 | N=4  N=11  N=9  N=1 | 16%  44%  36%  4% |  | N=4  N=11  N=9  N=1 | 16%  44%  36%  4% |  | 1.0 |

*Only in arteriovenous fistulas

ATA: Asymmetric cellulose triacetate

**Table S4: Post HD analytical data from the first HD session (session 1).**

|  | **ATA membrane** | | | **Synthetic membrane** | | |  |
| --- | --- | --- | --- | --- | --- | --- | --- |
|  | **N** | **media** | **DE** | **N** | **media** | **DE** | P value |
| **Hemoglobin (g/dl)** | **16** | 13 | 1.2 | **16** | 12.9 | 1.4 | 0.8 |
| **Leucocites (10^-3^/ul)** | **16** | 6.3 | 2.1 | **16** | 6.3 | 2.3 | 1.0 |
| **Neutrophils (10^-3^/ul)** | **15** | 4.3 | 1.9 | **16** | 4.2 | 2 | 0.9 |
| **Linfocites (10^-3^/ul)** | **15** | 1.3 | 0.4 | **16** | 1.3 | 0.5 | 0.9 |
| **Monocites (10^-3^/ul)** | **15** | 0.4 | 0.1 | **16** | 0.4 | 0.2 | 0.9 |
| **Eosinophils (10^-3^/ul)** | **15** | 0.2 | 0.2 | **16** | 0.2 | 0.2 | 0.3 |
| **basophils (10^-3^/ul)** | **15** | 0 | 0.1 | **16** | 0 | 0 | 0.1 |
| **Platelets (10^-3^/ul)** | **16** | 177.7 | 64.1 | **16** | 166.8 | 52.2 | 0.6 |
| **Glucose (mg(dl)** | **16** | 97.3 | 22 | **16** | 93.3 | 15.5 | 0.6 |
| **Urea (mg/dl)** | **16** | 37.5 | 57.5 | **15** | 23.5 | 12.1 | 0.4 |
| **Creatinin (mg/dl)** | **16** | 2.2 | 1.6 | **16** | 1.7 | 0.9 | 0.3 |
| **Sodium (mmol/l)** | **16** | 136.8 | 2.2 | **16** | 136.8 | 1.9 | 1.0 |
| **Potasium (mmol/l)** | **16** | 3.3 | 0.5 | **16** | 3 | 0.5 | 0.05 |
| **Total proteins (g/dl)** | **16** | 6.4 | 0.7 | **16** | 6.6 | 0.7 | 0.4 |
| **Albumin (g/dl)** | **16** | 3.8 | 0.5 | **16** | 3.9 | 0.5 | 0.7 |
| **Prealbumin (mg/dl)** | **15** | 21.2 | 11.9 | **15** | 20.5 | 12.1 | 0.9 |
| **β2M (mg/dl)** | **16** | 6.1 | 2.4 | **15** | 6.5 | 2.7 | 0.7 |
| **Mioglobin (ng/ml)** | **8** | 45.2 | 12.7 | **8** | 90 | 33.9 | 0.003 |
| **CRP (mg/l)** | **16** | 9.5 | 12.6 | **16** | 13.3 | 17 | 0.5 |
| **PTT (s)** | **15** | 29.9 | 10.4 | **16** | 31.8 | 12 | 0.6 |
| **PT (s)** | **15** | 12.6 | 1 | **16** | 12.2 | 0.9 | 0.6 |
| **INR** | **15** | 1 | 0.1 | **16** | 1 | 0.1 | 0.2 |
| **Anti factor XA (UI/ml)** | **8** | 0.2 | 0.1 | **8** | 0.2 | 0.1 | 0.1 |

*** *Data show mean and standard deviation. Student t-test was used to analyze the statistical differences. Missing data in some variables are due to errors during analytical extraction (missing requests, tube coagulation… etc.)*

β2M: Beta2 microglobulin; CRP: C-reactive protein; PTT: partial thromboplastin time; PT: prothrombin time.

**Table S5: Post HD analytical data from the last HD session (session 6).**

|  | **ATA membrane** | | | **Synthetic membrane** | | |  |
| --- | --- | --- | --- | --- | --- | --- | --- |
|  | **N** | **media** | **DE** | **N** | **media** | **DE** | P value |
| **Hemoglobin (g/dl)** | 14 | 12.7 | 1.6 | 6 | 12.4 | 0.9 | 0.3 |
| **Leukocytes (10^-3^/µl)** | 14 | 5.9 | 2.5 | 6 | 6.1 | 2.3 | 0.7 |
| **Neutrophils (10^-3^/µl)** | 13 | 3.6 | 2.5 | 5 | 4.4 | 2 | 0.9 |
| **Lymphocytes (10^-3^/µl)** | 13 | 1.4 | 0.3 | 5 | 1.2 | 0.3 | 0.3 |
| **Monocytes (10^-3^/µl)** | 13 | 0.4 | 0.2 | 5 | 0.4 | 0.1 | 0.7 |
| **Eosinophils (10^-3^/µl)** | 13 | 0.2 | 0.1 | 5 | 0.3 | 0.2 | 0.2 |
| **Basophils (10^-3^/ul)** | 13 | 0.1 | 0.2 | 5 | 0 | 0.1 | 0.5 |
| **Platelets (10^-3^/µl)** | 14 | 154.3 | 35.3 | 6 | 147.2 | 48.9 | 0.6 |
| **Glucose (mg/dl)** | 13 | 93.1 | 14.3 | 6 | 96.8 | 14 | 0.4 |
| **Urea (mg/dl)** | 11 | 17.7 | 8.2 | 4 | 21.5 | 10.5 | 0.2 |
| **Creatinin (mg/dl)** | 14 | 1.4 | 0.4 | 6 | 1.5 | 0.5 | 0.6 |
| **Sodium (mmol/l)** | 14 | 137 | 1.6 | 6 | 136.8 | 1.3 | 0.8 |
| **Potasium (mmol/l)** | 14 | 2.9 | 0.3 | 6 | 3.1 | 0.5 | 0.2 |
| **Total protein (g/dl)** | 14 | 6.7 | 0.6 | 6 | 6.5 | 0.6 | 0.5 |
| **Albumin (g/dl)** | 15 | 3.9 | 0.5 | 6 | 4 | 0.3 | 0.5 |
| **Prealbumin (mg/dl)** | 9 | 23.1 | 10.4 | 4 | 19.1 | 14.1 | 0.8 |
| **β2M (mg/dl)** | 13 | 6.2 | 3.1 | 5 | 5 | 1.3 | 0.5 |
| **Myoglobin(ng/ml)** | 8 | 54.3 | 11.1 | 3 | 62.3 | 40.4 | 0.07 |
| **CRP (mg/l)** | 14 | 5.6 | 10.8 | 6 | 5.6 | 7.1 | 0.9 |
| **PTT (s)** | 14 | 27.1 | 2 | 6 | 27.9 | 4.7 | 0.8 |
| **PT (s)** | 14 | 12.2 | 1.1 | 6 | 12.2 | 1.2 | 0.9 |
| **INR** | 14 | 1 | 0.1 | 6 | 1 | 0.1 | 0.7 |
| **Anti factor XA (UI/ml)** | 9 | 0.1 | 0 | 3 | 3.4 | 5.7 | - |

*** *Data show mean and standard deviation. Student t-test was used to analyze the statistical differences. Missing data in some variables due to errors during analytical extraction (missing requests. tube coagulation… etc.)*

β2M: Beta2 microglobulin; CRP: C-reactive protein; PTT: partial thromboplastin time; PT: prothrombin time.

**Table S6: PreHD and post HD analytical data comparing ATA membrane to synthetic membrane.**

|  | **PRE** | | | |  | **POST** | | | |  |
| --- | --- | --- | --- | --- | --- | --- | --- | --- | --- | --- |
|  | **N** | **ATA membrane** | **N** | **Synthetic membrane** | **P value** | **N** | **ATA membrane** | **N** | **Synthetic membrane** | **P value** |
| **Hemoglobin (g/dl)** | 15 | 11.8 (1.2) | 6 | 11.6 (0.7) | 0.78 | 14 | 12.7 (1.6) | 6 | 12.4 (0.9) | 0.73 |
| **Platelets (10^-3^/µl)** | 15 | 164.3 (42.9) | 6 | 170.5 (74.5) | 0.81 | 14 | 154.3 (35.3) | 6 | 147.2 (48.9) | 0.72 |
| **PTT** | 11 | 27.5 (2) | 6 | 27.6 (1.6) | 0.98 | 14 | 27.1 (2) | 6 | 27.9 (4.7) | 0.6 |
| **PT** | 11 | 13 (1.1) | 6 | 12.8 (1.4) | 0.73 | 14 | 12.2 (1.1) | 6 | 12.2 (1.2) | 0.99 |
| **Anti factor XA (UI/ml)** | 9 | 0.1 (0) | 3 | 0.1 (0) | 0.99 | 9 | 0.1 (0) | 3 | 3.4 (5.7) | 0.08 |
| **INR** | 11 | 1 (0.1) | 6 | 1 (0.1) | 0.38 | 14 | 1 (0.1) | 6 | 1 (0.1) | 0.75 |

PTT: partial thromboplastin time; PT: prothrombin time; INR: international normalized ratio.

Figure S1. Coagulation score.


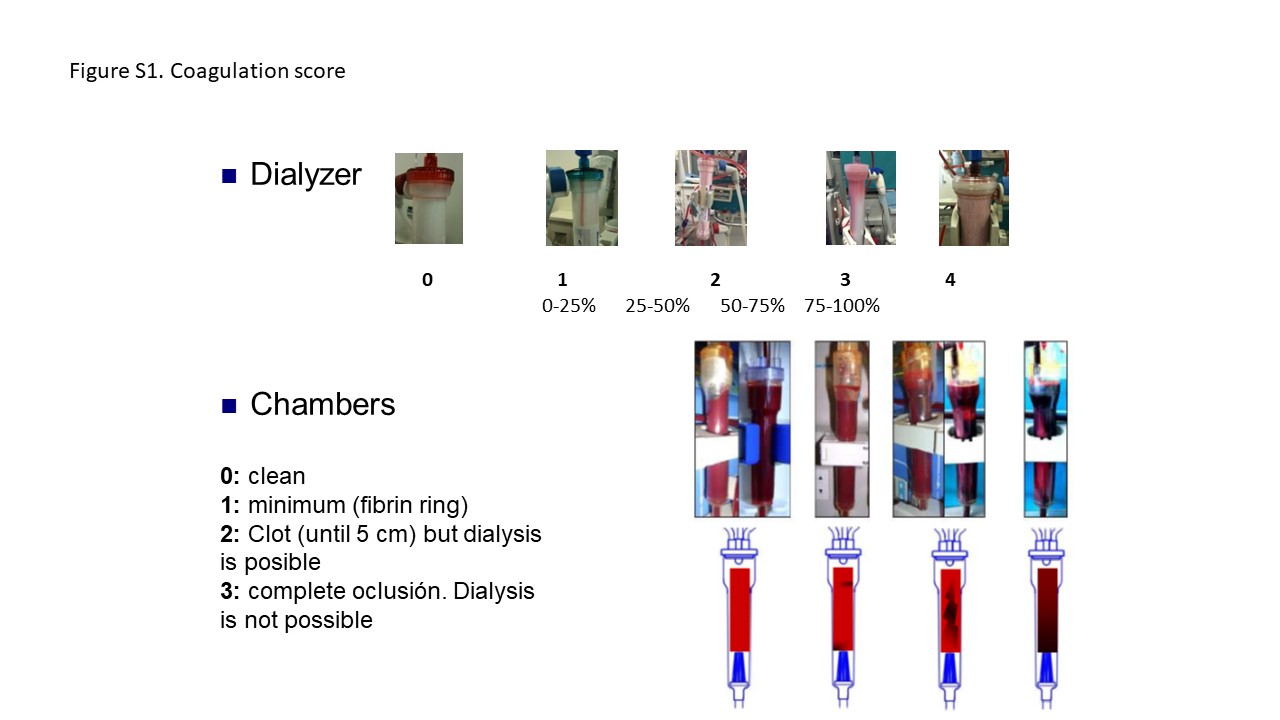


1. 20. de Sequera Ortiz P, Pérez García R, Molina Nuñez M, Muñoz González RI, Álvarez Fernández G, Mérida Herrero E, Camba Caride MJ, Blázquez Collado LA, Alcaide Lara MP, Echarri Carrillo R; en representación del grupo del estudio ABC-treat; Grupo del estudio ABC-treat. Prospective randomised multicentre study to demonstrate the benefits of haemodialysis without acetate (with citrate): ABC-treat Study. Acute effect of citrate. Nefrologia (Engl Ed). 2019 Jul-Aug;39(4):424-433. English, Spanish. doi: 10.1016/j.nefro.2018.11.002. Epub 2019 Jan 24. PMID: 30686542. [↑](#endnote-ref-2)
